# The Impact of Housing Insecurity on Access to Care and Services among People Who Use Drugs in Washington, DC

**DOI:** 10.3390/ijerph19137561

**Published:** 2022-06-21

**Authors:** Monica S. Ruiz, Allison Williams, Allison O’Rourke, Elizabeth MacIntosh, Shareese Moné, Cyndee Clay

**Affiliations:** 1 Department of Prevention and Community Health, Milken Institute School of Public Health, The George Washington University, 950 New Hampshire Ave. NW, Suite 300, Washington, DC 20052, USA; alliemwill@gwmail.gwu.edu; 2 DC Center for AIDS Research, Department of Psychological and Brain Sciences, 2125 G St. NW, Washington, DC 20052, USA; orourkea@email.gwu.edu; 3 Honoring Individual Power and Strength (HIPS), 906 H St. NE, Washington, DC 20002, USA; elizabeth@hips.org (E.M.); shareese.mone@gmail.com (S.M.); cyndeeclay@hips.org (C.C.)

**Keywords:** drug user, harm reduction, housing, access to care

## Abstract

People who use drugs are highly marginalized communities and are disproportionately affected by environmental changes—e.g., neighborhood gentrification—that affect housing availability and stability, particularly in urban locations. These changes could negatively affect individuals’ access to and utilization of health care and social services, resulting in poorer health outcomes. This study examined the impact of gentrification and housing instability on drug users’ access to harm reduction and other health services. Data were collected from 139 clients of a large harm reduction organization. Results showed that 67% of the participants were either unstably housed or homeless, and about one-third of participants indicated that their current housing situations negatively affected their access to primary care (33.9%), behavioral health services (36.7%) and basic services (38.3%). While homeless individuals were still able to access services generally, a greater percentage—compared to those unstably or stably housed—reported difficulty accessing care. As these data were collected prior to the COVID pandemic, it is likely that many of our participants faced greater struggles with housing insecurity and health care access issues due to shutdowns and increased need for social isolation and quarantine. More work is needed to address housing instability and homelessness among already marginalized populations.

## 1. Introduction

One of the inevitable processes occurring in large urban centers is that of gentrification, defined as “an interactive process in formerly declining, under-resourced, predominantly minority neighborhoods involving economic investment and increasing sources of capital infusion and in-migration of new residents, generally with a higher socio-economic status [1]”. Substantial research has been conducted on the effects of gentrification on the various economic and structural aspects of the urban landscape, such as how these changes affect neighborhood racial, ethnic and class composition and social displacement, etc. [2,3,4]. The issue of displacement has become a hallmark of the gentrification process, in that the transformation of neighborhoods that allows for the influx of more affluent businesses and residents may simultaneously force out long-time residents, particularly those who are already economically marginalized, to the peripheries of urban centers and away from existing support networks [5]. This displacement may occur in a number of ways, including being priced out of a dwelling through rent increases or by physical means (i.e., economic/physical displacement) [6] or being unable to access property because it has been gentrified (i.e., exclusionary displacement) [6].

One of the most notable effects of gentrification is the impact that it has on the communities that are displaced through the process. Research and history have shown that economically disadvantaged peoples—including but not limited to the elderly, persons from racial and ethnic minority backgrounds, etc.—are disproportionately driven (by poverty, structural racism, discriminatory housing policies, redlining, etc.) to live in under-resourced neighborhoods [7,8,9,10]. Because these same neighborhoods are the ones targeted for gentrification and urban renewal, these same communities of people are disproportionately represented among those who have been displaced by the process [11]. For this reason and keeping in mind the co-occurrence of economic disparity with other types of social and political disparity, gentrification has often been considered a form of structural violence, i.e., a construct that describes the ways in which the socio-political and economic organization of the society visibly and systematically fosters harm to vulnerable groups within that society [12,13,14].

While displacement is not always considered negative or disruptive [15,16], there are certain impacts that cannot be ignored. For example, Ding et al. found that while economically disadvantaged residents of gentrifying neighborhoods did not always relocate, those that did often relocated to poorer neighborhoods [17], potentially exacerbating cycles of poverty and disparity. Additionally, gentrification may affect health outcomes among those who are displaced. A systematic review of the impacts of gentrification on health found that, while the impacts of gentrification are not uniform across all populations, historically marginalized populations are more likely to be affected compared to historically privileged populations [18] and may worsen existing health inequities [9]. To that end, it is important to examine the shorter- and longer-term health effects of gentrification and other forms of urban renewal on those already experiencing structural violence.

The District of Columbia has been undergoing significant urban transformation since the early 2000s, resulting in the gentrification of 54 neighborhoods [19]. It was one of the cities with the highest rate of gentrification between 2000 and 2013 and, along with six other cities (New York City, Los Angeles, San Diego, Philadelphia, Baltimore and Chicago), contributed to nearly half of the total gentrification nationally [20]. While this may have had the result of increasing the economic status of the city overall through higher property values and more competitive rental markets, it also had the effect of displacing 20,000 African American residents [20] and disrupting the provision of services through the closing or relocation of business that are trusted and relied upon by those communities [21]. 

While some research has been conducted on the effect of gentrification on populations in the District [22], less is known about how it has affected highly vulnerable populations, such as persons who use drugs (PWUD). In the District, this is served by a network of harm reduction organizations that provide syringe access and other harm reduction services through fixed site and mobile outreach. According to our 2016 population estimate, there were 8829 (95% CI 4899 and 12,759) PWUD who stated that they lived in Washington, DC. [23] The majority of these individuals were 40 years of age or older (68.2%) and self-identified as Black/African American (83.9%) [23]. While the personal contexts of the individuals within this population vary, many of these individuals had long-term lived experience with substance use and addiction, with 62.8% having initiated injection drug use before the age of 20 and 20.5% having initiated drug use between the ages of 20 and 29 [23]. At the time that those data were collected, the circumstances of individuals’ housing status were not assessed, so it is possible that there were persons in that population who were both stably housed and unstably housed/unhoused.

Because many in this population are also experiencing severe economic disparity, housing stability is a critical and ongoing issue, particularly as it pertains to the maintenance of health status and the avoidance of negative health outcomes. There is substantial evidence confirming that lack of stable housing poses a barrier to accessing appropriate health care services, including HIV care [24] and behavioral health treatment [25]. To that point, social policies such as gentrification and their associated effects—forced relocation due to unaffordable housing costs, closure or relocation of existing neighborhood services and dissipation of community support systems—can be a significant barrier to PWUD, not only in terms of exacerbating economic disparity and instability but because they may affect access to necessary medical, social and harm reduction services. Consistent access to harm reduction services is critical for the maintenance of health behaviors that can help reduce infection with HIV and other bloodborne diseases, including hepatitis B/C and endocarditis. For many PWUD, having such services close to their place of residence is critical; increases in distance and travel time to services can result in reduced engagement with providers and poor retention in care [26]. To that end, it is important to know how gentrification is affecting this population, so that service provision can be modified to meet their changing needs. The purpose of this exploratory cross-sectional study is to examine the impact of gentrification on the ability of DC PWUD to access harm reduction and other health-related services. 

## 2. Materials and Methods

This study was a research collaboration between the Milken Institute School of Public Health, George Washington University, and Honoring Individual Power and Strength (HIPS), a non-profit harm reduction organization in Washington, DC, which provides harm reduction and supportive services to persons and communities affected by sex work and substance use. 

### 2.1. Participant Recruitment

Participants for this study were recruited from HIPS’ clients who were presenting for services at syringe exchange sites or the drop-in center or engaging with harm reduction outreach workers at community-based venues (e.g., parks, shelters, etc.). Individuals who were eligible for study participation were over the age of 18 and had to be capable of and willing to provide verbal informed consent for study participation.

If individuals were willing to participate in the study, verbal consent was obtained and documented by a graduate student interviewer with extensive prior experience working with harm reduction organizations and vulnerable populations served by such organizations. At no point during the data collection period did the interviewer encounter a participant who was unable to provide meaningful answers to survey questions, so all participants were included in the analyses.

Surveys were developed for online completion using Qualtrics; the interviewer administering the survey was able to enter the participants’ responses into the online instrument. All interviews were completed in semi-private locations within the HIPS offices or in the community (e.g., a location away from others). Following survey completion, each participant was given a USD 10 gift card in appreciation of their time. This amount was determined by HIPS staff as being appropriate for the population. Participants were also given a Study Information sheet, which contained information on the purpose of the study, the human subjects’ protection measures being taken for the project, the IRB approval number for the research project and the telephone numbers for the GWU Office of Human Research and the PI. This research was approved by the George Washington University Institutional Review Board (IRB# 021854).

### 2.2. Measures

#### 2.2.1. Demographics

Demographic measures included self-identified age, race, ethnicity, sex assigned at birth, current gender identity and current substance use behavior. Race was broken down into five categories: Black/African American, Caucasian/White, Asian/Pacific islander, Native American/Alaska Native and other. Recent (3 months prior to survey) substance use measures asked if the participant had used any drugs/substances in the past 3 months (yes/no), if any of these substances were prescribed (yes/no) and if the participant had injected drugs in the previous 3 months (yes/no).

#### 2.2.2. Housing Stability

Housing stability was assessed with two questions, one focusing on housing in the previous two years and the other assessing current housing status. Participants were asked to think about their housing situation in the prior 2 years and to self-identify into one of four categories: stably housed (defined as they have always had a place to live), mostly stably housed (defined as they have had a place to live most of the time but were sometimes homeless), mostly unstably housed (defined as they were homeless most of the time or did not have a stable place to live), or homeless the entire time in the prior 2 years. Participants were then asked to identify from three choices which one best described their current level of housing stability: homeless, not a stable place (unstably housed) or stably housed. For this paper, current housing stability is used for data reporting and analyses.

If participants indicated that they were stably housed, they were asked to indicate how long they have lived in their current location, the type of housing in which they were living, with whom they were living (living arrangement) and reason for living in this location. Housing type categories included owning a place or renting off the market, living with a friend/family, public/subsidized housing, transitional living and other. Living arrangement categories included lives alone, lives with a partner/significant other, lives with family, lives with friends, lives with people who are not friends and other. Participants were also asked to indicate qualities about their current place of residence that made it desirable, such as being affordable, closer to services or work opportunities, safety and free to stay. The stability of the living situation was assessed through questions asking about how long participants felt they could stay at that location and their self-assessed risk about potentially losing this living situation. They were also asked about why they left/moved from the living situation prior to the current one.

Participants who indicated that they were currently unstably housed were asked similar questions regarding the length of time in their current living situation, the type of housing it was, with whom they were currently living and the qualities about their current place of residence that made it desirable. However, these participants were also asked several additional questions to better understand why their housing situation was unstable, including how long it had been since they lived in stable housing and the timeline for their current living arrangement. Unstably housed participants were also asked about the place where they lived prior to the current location and why they left that location.

Participants who indicated that they were homeless were asked how long they had been homeless and the reasons that caused them to become homeless. They were then asked about the place they lived in immediately prior to becoming homeless, including length of time and the type of housing it was.

For all the housing-related questions, the survey used for the study provided some possible response options to the questions. These response options were created by the research team but were largely informed by HIPS’ staff and their experiences of working with the participant population. Understanding that people’s lives are complex, participants were encouraged to pick as many responses in those sets of response options as they felt represented their experience. Additionally, participants were encouraged to provide qualitative information to elaborate on their lived experiences.

#### 2.2.3. Access to Services

Participants were asked about where they were currently living and whether or not this location negatively affects their ability to physically access a variety of essential services, including syringe exchange/distribution services, medical/primary care services, behavioral health care services (including mental health services, medication-assisted treatment for substance use disorder, etc.), social services (e.g., community drop-in centers, etc.) and basic services (e.g., laundry services, food access, etc.). In this study, “access” was operationalized as the individual’s ability to physically get to a specific location/provider to receive care or services. The response format for these items allowed respondents to indicate that: (a) their current location did not affect their access; (b) yes, there was a negative impact, but they could still access services; (c) yes, there was a negative impact, and they could not access services; or (d) there was no impact because they did not use these services. In addition to examining these variables with each of the four response options, we also examined this by creating a dichotomous variable that compared no impact on access (answering no impact on the above items) with any impact on access (combining the two “yes” responses). Those indicating that they did not use the services in question were excluded from the analyses using the dichotomous variable.

Finally, respondents were invited to share thoughts they had about their current housing situation and how it affected their access to services. This open-ended query provided the opportunity for participants to contribute qualitative data pertaining to their own lived experiences with housing and the various social and economic factors that have affected their abilities to access important care and services.

### 2.3. Analyses

As this was an exploratory study, all data collected were cross-sectional in nature. Statistical analyses were limited to bivariate analyses to examine housing status and access to services. Chi-square analyses were conducted to compare access to services measures, comparing those who were stably housed to those who were unstably housed, and stably housed to homeless individuals. All quantitative analyses were conducted using SAS v9.4.

Qualitative data were collected to allow participants to share their thoughts about their own experiences as to how they are affected by gentrification and other changes to the urban landscape. These data serve the purpose of providing additional context to the quantitative data, and participant quotes are used in this paper to give voice to participants’ lived experiences with housing instability.

## 3. Results

### 3.1. Sample Demographics

Data collection began in the summer of 2019 and ended in March 2020, when the emergence of the COVID-19 pandemic forced all businesses and in-person activities to shut down for safety. At the time that the study was halted, 139 participants had been recruited into the study (approximately 56% of the target sample size of 250) and had completed surveys and interviews. Demographics are reported in Table 1. Participants ranged in age from 19 to 65 years, with the mean age being 40.3 years. Regarding self-identified gender, 47.5% of the sample identified as male, and an equal percentage identified as female, with three participants indicating a nonbinary gender identity. The majority (82%) indicated that their gender identity corresponded with the gender to which they were assigned at birth. Regarding race and ethnicity, 5.1% self-identified as Hispanic/Latinx, and 87.6% self-identified as Black/African American. The majority (79.0%) of the participants indicated that they had used some sort of substance in the 3 months prior to the survey. 

Regarding current housing status, only 33% of the participants self-identified as being stably housed, with the remainder self-identifying as being either unstably housed (23%) or homeless (44%). When asked about their general housing status in the two years prior to the survey, 37.3% indicated that they had been stably (23.2%) or mostly stably (13.8%) housed, 26.1% indicated that they had been unstably housed, and 37.0% indicated that they had been homeless the entire time.

### 3.2. Housing Status and Residential Instability: Comparisons between Groups

A deeper examination of participants’ current and prior housing status yielded more nuanced information regarding people’s housing stability and reasons for changes in housing. Table 2 reports on housing situation measures for those reporting being currently stably or unstably housed. Those who were unstably housed had been experiencing housing instability for varied lengths of time, ranging from less than 1 year (25.8%) to over 5 years (29.0%). Those who were stably housed had lived in their current locations for lengths of time ranging from less than 1 year (35.6%) to over 5 years (24.4%), whereas the majority of those unstably housed (87.5%) indicated that they had been in their current unstable housing situation for 2 years or less. While individuals in both groups lived in different types of housing, those who were stably housed compared to those who were unstably housed were more likely to own or be renting their current place (33.3% vs. 3.1%, respectively) and were more likely to be living alone (44.4% vs. 15.6%, respectively). Conversely, stably housed persons compared to those who were unstably housed were less likely to be in transitional living spaces (2.2% vs. 15.6%, respectively) or spaces defined as “other” (0% vs. 18.8%) and were less likely to indicate living in a situation with people who they did not consider friends (0% vs. 25.0%). 

Similarly, there were differences in individuals’ reasons for living in their present location. Those who were stably housed cited safety (45.5%), affordability (38.7%), proximity to services (34.1%) and proximity to work opportunities (25.0%) as their main reasons for their present location. While 20 individuals chose “other” as the response to the question regarding reasons for living in their current location, 24 individuals provided responses to the open-ended question seeking more clarification about that response. Of these 24 individuals, 29.2% (*n* = 7) cited environmental characteristics of the location (e.g., “quiet neighborhood”, “safer neighborhood”, etc.) as the reason for choosing that living location. Other popular responses were amenities or convenience of the location (*n* = 5, 20.8%), affordability of the location (either through housing vouchers or insurance) (*n* = 5, 20.8%) and the qualities of the relationship with the person with whom they were living (*n* = 5, 20.8%) as reasons for choosing that location. Most stably housed persons (95.8%) indicated that they were not in danger of losing their housing and could stay there as long as they wanted to (83.3%) or as long as they could pay the rent (12.5%).

In contrast, those who were unstably housed cited safety (31.3%) and lack of choice (“the only thing available”; 31.3%) as the top reasons for choosing their current locations. Among those who cited “other” reasons for living in their current location, 10 (66.7%) stated reasons that spoke to a combination of availability of housing and personal necessity for that type of housing. For instance, one participant who was currently staying at a shelter indicated that the shelter where they had stayed immediately prior to the current location had men and women housed together and also allowed clients to bring in their dogs. Such shelter situations were not ideal for the participant, as they were concerned about privacy as well as allergic to and afraid of dogs. Thus, they left that shelter and chose a different shelter because it had availability and was closer to an area they preferred. 

Among the unstably housed persons, less than half (41.9%) indicated an open-ended timeline for staying in their current location, with over a third (42.0%) saying that they would need to leave immediately or soon. When asked about the reasons for needing to leave their present housing location, participants cited interpersonal issues (*n* = 7; 21.9%) and concern about outstaying their welcome (*n* = 6; 18.8%) as reasons why they would leave. Of the 19 participants who chose the “other” response option, 36.8% (*n* = 7) indicated that they would leave their present situation if they found stable housing. Another 26.3% (*n* = 5) indicated that they would leave if the situation became unsafe for them to stay (e.g., being bullied because the individual is transgender), and another 21.0% (*n* = 4) indicated that there was an existing timeline and that they were scheduled to leave soon. Three other individuals (15.8%) stated reasons that could not be categorized because they were unsure of where they were staying (i.e., the individual had just arrived to the city) or were not sure of what would cause them to leave.

We also asked stably and unstably housed participants about their housing situation prior to their current location and, more specifically, asked for information about why they needed to leave that location. Participants were able to select more than one response if they desired. Only half of the stably housed participants provided information for this item and, of these, the majority cited family reasons for why they were forced to leave. Conversely, the unstably housed participants were more likely to cite numerous reasons for why they left their prior residence, including dissolution of a relationship (15.6%) and the location becoming unsafe (15.6%). 

Many unstably housed persons cited “other” reasons for needing to leave their prior place of residence and provided a wide variety of qualitative responses to explain. Of the 21 individuals who chose this category, 4 (19%) specifically stated that they left their prior place of residence to try to access housing services (e.g., by seeking services at a shelter, applying for housing vouchers, etc.). Three individuals (14%) indicated that their prior housing situation was that they were homeless, so the change was that they went from living on the street to living with someone else even if that situation was currently unstable. Another three individuals (14%) cited changes in the circumstances surrounding the person with whom they had been living as the reason for leaving their prior place of residence. Only three individuals cited reasons related to urban development and possibly gentrification, including the inability to afford rent after the buildings in which they had been living were sold to new owners (*n* = 2, 9.5%) and actions taken by the city to relocate homeless individuals from gentrifying areas (e.g., city “clean ups” occurred, and the individual’s tent and all their belongings were taken) (*n* = 1, 4.8%). Two individuals (9.5%) mentioned that their prior place of residence had been a correctional facility and that their term of incarceration ended. Other reasons provided pertained to changes in the individual’s personal life, such as changes in relationship status (e.g., either wanting to move in with or away from a partner) or health-related transitions (e.g., completing a treatment program and not being able to access transitional housing). One young participant indicated that they were unstably housed because the school year ended, and they needed to move out of their dormitory. 

In addition to these two groups, there were 60 individuals who indicated that they were currently unhoused or homeless. Their data are presented in Table 3. Overall, 63.7% of these participants had been homeless for 3 years or longer, but 68.3% had known some housing stability, in that they had lived in their prior location for 3 years or longer. Most individuals indicated that they had lived somewhere in the District of Columbia, with 28.3% indicating that their prior location of residence was outside of Washington, DC. The majority had either owned or rented their prior residence (25.0%) or lived with a friend or family member (48.3%). Participants cited several reasons for becoming homeless, including changes in personal economic situation (31.7%), situations related to urban development and possibly gentrification (21.7%) and changes in familial or romantic relationships (33.3%). Participants’ reasons for leaving their prior place of residence largely mirrored their reasons for being homeless. 

While 70% (*n* = 42) of the participants chose the “other” category when asked for reasons that led to their homelessness, 49 individuals provided information to the open-ended question that asked for more information. Their myriad responses provided a more nuanced perspective on how difficult it can be for individuals already struggling with poverty to maintain secure housing. Of the 49 respondents, 18.7% (*n* = 9) cited changes in familial situations (e.g., parental divorce, death in the family) as the reason for their homelessness. For example, one individual stated that they had been caring for an ill family member but were unable to stay in the home after that family member had passed due to seizure of the property by the government. Another individual cited a similar instance of taking care of an ill family member but becoming homeless when they were unable to transfer the deceased person’s housing voucher into their name. Release from incarceration and having no place to stay (*n* = 7; 14.3%) and untreated mental health or substance use issues (*n* = 7; 14.3%) were also commonly given reasons for homelessness. Six individuals (12.2%) cited becoming homeless because of disputes with the owners of their prior residence, including landlords who did not keep the properties up to code. Five individuals (10.2%) indicated that they were homeless because they could not afford market prices for housing, and four others (8.2%) cited changes in circumstances (such as disputes, etc.) with the persons with whom they had been living as the reason for their homelessness. Other responses included being homeless because of eviction from prior housing because of job loss (*n* = 3; 6.1%) and because of escaping domestic violence (*n* = 1, 2.0%).

### 3.3. Impact of Housing Instability on Access to Services

We then asked participants if their current housing situation negatively affected access to a variety of services using the dichotomous variable described earlier. Chi-Square tests were used to examine statistically significant differences, using stably housed individuals as the referent group. These data are presented in Table 4. About one-third of participants across all five service categories indicated that their living situation negatively affected their access. When compared across housing categories, we found significant differences between the stably housed and homeless individuals for accessing four of the five service categories (medical/primary care, other health services, basic service and other supportive services) but no differences between those stably housed and unstably housed. 

Several participants noted that gentrification in the District of Columbia has also affected the presence of services in the city generally, especially in areas where gentrification drives up business rental property, forcing smaller service-providing organizations to move or close. Such changes added to the confusion about the availability of services among marginalized communities, especially if such changes and closures were not documented or updated regularly and in a central location. As one participant noted,

*“So many changes in Washington DC since I grew up here. There used to be advocates for services so that people knew exactly where to go for what they needed. Now, between all the new buildings and new locations, it’s difficult to figure out where services are. People use Facebook for a lot of information, but it’s often not available through government websites, for example. Businesses and organizations have to move because they can’t afford the rent and then no one knows where they went or how to access their services”*.

## 4. Discussion

These data provide a preliminary examination of the impact of housing status on access to medical, behavioral health, harm reduction and other social services in the District of Columbia. We found that housing status did have a significant impact on individuals’ access to care, in that people who were stably housed were generally able to reach the services they needed, while those unstably housed or homeless reported significantly more difficulty accessing services. Reasons for experiencing homelessness were largely due to individuals’ personal circumstances changing and no longer being able to afford previous housing.

One significant limitation of this research is the sample size, which was directly related to the COVID-19 pandemic. Our target sample size was 250 individuals, but we were only able to recruit and collect data from 139 participants before pandemic shutdowns occurred. This not only precluded the ability to complete the study as planned but also forced the cancelation of collecting more qualitative data from focus groups that had been planned for March and April of 2020. While we were able to perform descriptive analyses, the smaller sample size did not allow for the performance of more detailed analyses to examine how gentrification may have affected participants’ lives. Another issue is that of service utilization by our participants. Because we asked about participants’ current usage rather than their usage in the prior 6 months or year, our findings may underestimate individuals’ patterns of services utilization or their need of services. 

Despite the limitations, these findings indicate that more research is needed to understand how urban transformation processes, such as gentrification, affect already marginalized populations. While we did obtain important data about the reasons why individuals suffer from housing insecurity, many of the reasons that participants gave for their housing insecurity did not expressly speak to forced relocation due to gentrification. Instead, our data revealed a variety of other personal circumstances that may contribute to difficulties in securing housing. These circumstances include being forced to live with family or friends instead of being able to live on one’s own, having difficulty finding housing that provides safety (e.g., from domestic violence or homophobic or transphobic environments), trying to cope with untreated behavioral health issues and having trouble navigating governmental bureaucracies to receive housing assistance. In addition to these challenges, gentrification may pose an additional burden in terms of finding housing in a market that is largely unaffordable to already socially and economically marginalized individuals. As one participant stated,

*“Without housing, how can anyone do anything? Housing gives you stability. If you have nowhere to sleep at night, it’s hard to do any of the basic things you need to do to take care of yourself. Lack of housing is lack of support and everything else. If they’re only building condos, it’s hard to get housing in DC. We couldn’t afford it when it was a thousand dollars, now it’s $2500 and we still can’t afford it”*.

Another issue that warrants further investigation are the cycles of disparity that perpetuate long-term housing insecurity and instability. Approximately one-third of our participants who described themselves as unstably housed had been in this situation for 5 or more years. Moreover, many of those unstably housed persons who currently had a place to stay indicated that they were living with friends or family. While this may be a viable short-term solution, it may not be feasible for individuals to live in this fashion for longer periods of time, which further contributes to the instability of their housing situation. Additional research is needed to examine not only why individuals are unstably housed but how resources can be provided to break the cycles of disparity, so that individuals can have stable, sustainable and safe housing. 

While quantitative analyses were hampered by the smaller sample size, the substantial amount of qualitative data provides the opportunity to better understanding the complex needs of the PWUD population, particularly as these needs intersect with the various city agencies with whom people need to work to obtain supportive services. This was particularly true regarding the final open-ended question in our survey, which provided participants the opportunity to share any last thoughts with the study team. Thirty participants specifically mentioned negative experiences with city agencies, with housing services being the most common target (e.g., poor communication/unresponsiveness, disrespect to the participant, etc.). Homeless shelters were another frequent subject of complaint, with participants sharing experiences of discrimination, drug use, theft and violence. While city officials have made some progress on the plan to end homelessness in the District by 2025 [27], these efforts are falling short of their goal and are having trouble addressing the complex needs of the marginalized and vulnerable populations—such as PWUD—who need housing, health care and other supportive services [28]. Our data not only provide insight about drug users’ general housing needs but also the difficulties they have encountered trying to work with the systems that too frequently fail them. Efforts to improve the quality of services for unstably housed and homeless PWUD should take care to better understand the lived experiences of PWUD, as well as ensure that services are provided in a manner that fosters trust, dignity and respect.

## 5. Conclusions

This research found that housing instability did affect access to medical and other health and social services among city residents. More research is needed to better understand how urban development processes, such as gentrification, affect the ability of marginalized populations to navigate linkage to and retention in health care services, particularly in contexts where housing is unstable or not available. Further research is needed to better understand the mechanisms that contribute to and strengthen the resilience of marginalized populations and the organizations that serve them to optimize efforts for medical and social service provision. Additionally, our data speak to the need for substantial improvements in programs to address housing insecurity among PWUD, as well as the other complex needs of this population.

## Figures and Tables

**Table 1:** Sample demographics (*n* = 139).

Variable	*n* (%)
Age	
• ≤29	29 (20.9)
• 30 to 39	52 (37.4)
• 40 to 49	19 (13.7)
• 50 to 59	27 (19.4)
• 60 to 69	12 (8.64)
Self-identified race	
• Black/African American	120 (87.6)
• Caucasian/White	15 (11.0)
• Asian/Pacific islander	2 (1.5)
• Native American/Alaska Native	26 (19.0)
• Other	11 (8.0)
Self-identified Hispanic/Latinx	7 (5.1)
Self-identified gender	
• Male	66 (47.5)
• Female	65 (46.8)
• Gender Nonbinary	4 (2.9)
• Other	4 (2.9)
Self-identified gender corresponds to sex assigned at birth	114 (82.0)
Self-reported substance use	
• Substance use in the past 3 months	109 (79.0)
• Injection drug use	21 (19.3)
• Use of prescribed drugs	12 (11.1)
Self-described housing situation in prior two years	
• Stably housed	32 (23.2)
• Mostly stably housed	19 (13.8)
• Unstably housed	36 (26.1)
• Homeless	51 (37.0)
Current housing status	
• Stably housed	46 (33.3)
• Unstably housed/Not living in a stable place	32 (23.2)
• Homeless	60 (43.5)

**Table 2:** Descriptions of housing situations for those who are stably and unstably housed.

	Current Housing Status	
Variable	Stably Housed (*n* = 45)	Unstably Housed (*n* = 32)
Length of time since stably housed		
• <1 year	-	8 (25.8)
• 1–2 years		7 (22.6)
• 3–5 years		7 (22.6)
• >5 years		9 (29.0)
Length of time at current housing location		
• <1 year	16 (35.6)	19 (59.4)
• 1–2 years	9 (20.0)	9 (28.1)
• 3–5 years	9 (20.0)	3 (9.4)
• >5 years	11 (24.4)	1 (3.1)
Type of Housing (current)		
• Own a place or rent off the market	15 (33.3)	3 (9.3)
• Live with a friend/family	12 (26.7)	16 (50.0)
• Public/subsidized housing	17 (37.8)	7 (21.9)
• Transitional living	1 (2.2)	6 (18.8)
• Shelter	0	4 (12.5)
• Public space (e.g., a parking garage)	0	1 (3.1)
Co-habitation/living with others		
• Lives alone	20 (44.4)	5 (15.6)
• Lives with a partner/significant other	8 (17.8)	7 (21.9)
• Lives with family	14 (31.1)	6 (18.8)
• Lives with friends	8 (17.8)	7 (21.9)
• Lives with people who are not friends	0	8 (25.0)
• Other	1 (2.2)	0
Reason for living in this location		
• Only thing available	7 (15.9)	10 (31.3)
• Partner and I wanted to live together	2 (4.6)	4 (12.5)
• More affordable	17 (38.7)	2 (6.3)
• Closer to services	15 (34.1)	4 (12.5)
• Closer to work opportunities	11 (25.0)	3 (9.4)
• Safer location	20 (45.5)	10 (31.3)
• Free place to stay	0	4 (12.5)
• Other	20 (45.5)	15 (46.9)
Stability of current location of residence		--
• I can stay as long as I need or want to	20 (83.3)	
• I can stay here as long as I can pay rent	3 (12.5)	
• I can stay here for a while longer	0	
• I have to leave soon	0	
• Other	1 (4.2)	
Worry about losing the current living space		--
• Not at all	20 (83.3)	
• A little bit	2 (8.3)	
• Sometimes	1 (4.2)	
• Frequently	1 (4.2)	
• All of the time	0	
Currently at risk of losing this housing		--
• No	23 (95.8)	
• Maybe/not sure	1 (4.2)	
• Yes	0	
What would be cause for leaving this location?	--	
• Nothing would cause me to leave		5 (15.6)
• Financial considerations		1 (3.1)
• Worry about outstaying my welcome		6 (18.8)
• Interpersonal issues		7 (21.9)
• Other		19 (59.4)
Timeline for this arrangement	--	
• Open ended		13 (41.9)
• Need to leave in the future		5 (16.1)
• Need to leave soon		11 (35.5)
• Need to leave immediately		2 (6.5)
Length of time at prior location of residence		
• <1 year	13 (29.6)	12 (37.5)
• 1–2 years	14 (31.8)	8 (25.0)
• 3–5 years	8 (18.2)	3 (9.4)
• >5 years	9 (20.5)	9 (28.1)
Type of Housing (prior)		
• Own a place or rent off the market	11 (25.0)	4 (12.5)
• Live with a friend/family	14 (31.8)	7 (21.9)
• Public/subsidized housing	5 (11.4)	2 (6.3)
• Transitional living	2 (4.6)	3 (9.4)
• Homeless shelter	6 (13.6)	2 (6.3)
• Other	6 (13.6)	14 (43.8)
Co-habitation/lived with others		
• Lived alone	9 (20.5)	12 (37.5)
• Lived with a partner/significant other	7 (15.9)	4 (12.5)
• Lived with family	15 (34.1)	9 (28.1)
• Lived with friends	8 (18.2)	4 (12.5)
• Lived with people who are not friends	8 (18.2)	7 (21.9)
• Other	1 (2.3)	0
Why individual left prior location of residence		
• Relationship broke down	2 (4.7)	5 (15.6)
• Neighborhood changed; could no longer afford it	2 (4.7)	3 (9.4)
• Circumstances changed; could not afford payments	4 (9.3)	3 (9.4)
• Evicted/kicked out	3 (7.0)	2 (6.3)
• Was no longer safe to stay here	4 (9.3)	5 (15.6)
• Too far from services	0	1 (3.1)
• Too far from work opportunities	0	1 (3.1)
• Family reasons	5 (11.6)	2 (6.3)
• Other	0	21 (65.6)

**Table 3:** Description of housing instability among unhoused participants (*n* = 60).

Variable	*n* (%)
Length of time of being homeless	
• <1 year	11 (18.3)
• 1–2 years	11 (18.3)
• 3–5 years	13 (21.7)
• over 5 years	25 (41.7)
Cause of homelessness	
• Relationship broke down	11 (18.3)
• Neighborhood changed; could no longer afford it	3 (5.0)
• Circumstances changed; I could not pay what I was paying	23 (38.3)
• Evicted/kicked out	11 (18.3)
• Was no longer safe to stay here	10 (16.7)
• Family reasons	15 (25.0)
• Other	42 (70.0)
Length of time at prior residence	
• <1 year	8 (13.3)
• 1–2 years	11 (18.3)
• 3–5 years	14 (23.3)
• >5 years	27 (45.0)
Type of Housing (prior residence)	
• Owned a place or rented off the market	15 (25.0)
• Lived with a friend/family	29 (48.3)
• Public/subsidized housing	5 (8.3)
• Transitional living	1 (1.7)
• Homeless shelter	4 (6.7)
• Other	6 (10.0)
Co-habitation/living with others at prior residence	
• Lived alone	11 (18.6)
• Lived with a partner/significant other	12 (20.3)
• Lived with family	30 (50.8)
• Lived with friends	9 (15.3)
• Lived with people who are not friends	4 (6.7)
• Other	0
Why individual left this place	
• Relationship broke down	12 (20.0)
• Neighborhood changed; could no longer afford it	1 (1.7)
• Circumstances changed; I could not pay what I was paying	16 (26.7)
• Evicted/kicked out	10 (16.7)
• Was no longer safe to stay here	7 (11.7)
• Too far from services	1 (1.7)
• Too far from work opportunities	1 (1.7)
• Family reasons	15 (25.0)
• Other	31 (51.7)

**Table 4:** Access to services among participants according to housing status.

Variable	Total Responding *n* (%)	Stably Housed *n* (%)	Unstably Housed *n* (%)	Homeless *n* (%)
Current living situation negatively affects access to…				
• Medical or Primary care (*n* = 130)	44 (33.9)	10 (22.7)	7 (15.9)	27 (61.4) *
• Other health services (*n* = 90)	33 (36.7)	6 (18.2)	4 (12.1)	23 (69.7) *
• Syringe access/exchange or other harm reduction services (*n* = 34)	7 (20.6)	1 (14.3)	0 (0)	6 (85.7)
• Basic services (e.g., food, laundry, etc.) (*n* = 120)	46 (38.3)	6 (13.0)	9 (19.6)	31 (67.4) *
• Other supportive services (e.g., outreach, drop-in, etc.) (*n* = 90)	28 (31.1)	4 (14.3)	5 (17.9)	19 (67.9) *

* Chi-square test comparing to stably housed, *p* < 0.05.

## Data Availability

The data supporting the results reported in this paper are in the possession of the Principal Investigator.

## References

[1] Tulier M.E., Reid C., Mujahid M.S., Allen A.M. (2019). “Clear Action Requires Clear Thinking”: A Systematic Review of Gentrification and Health Research in the United States. Health Place.

[2] Newman K., Wyly E.K. (2006). The Right to Stay Put, Revisited: Gentrification and Resistance to Displacement in New York City. Urban Stud..

[3] Shaw K.S., Hagemans I.W. (2015). ‘Gentrification Without Displacement’ and the Consequent Loss of Place: The Effects of Class Transition on Low-Income Residents of Secure Housing in Gentrifying Areas. Int. J. Urban Reg. Res..

[4] Wyly E.K., Hammel D.J. (2004). Gentrification, Segregation, and Discrimination in the American Urban System. Environ. Plan. Econ. Space.

[5] Atkinson R., Australian Housing and Urban Research Institute (2011). Gentrification and Displacement: The Household Impacts of Neighbourhood Change.

[6] Marcuse P. (1985). Gentrification, Abandonment, and Displacement: Connections, Causes, and Policy Responses in New York City. J Urb. Contemp. Law.

[7] Gee G.C., Ford C.L. (2011). Structural Racism and Health Inequities: Old Issues, New Directions. Bois Rev. Soc. Sci. Res. Race.

[8] LaVeist T.A. (1993). Segregation, Poverty, and Empowerment: Health Consequences for African Americans. Milbank Q..

[9] Cole H.V.S., Mehdipanah R., Gullón P., Triguero-Mas M. (2021). Breaking Down and Building Up: Gentrification, Its Drivers, and Urban Health Inequality. Curr. Environ. Health Rep..

[10] Smith R.J., Lehning A.J., Kim K. (2018). Aging in Place in Gentrifying Neighborhoods: Implications for Physical and Mental Health. Gerontologist.

[11] Fullilove M.T., Wallace R. (2011). Serial Forced Displacement in American Cities, 1916–2010. J. Urban Health.

[12] Farmer P. (2004). An Anthropology of Structural Violence. Curr. Anthropol..

[13] DeVerteuil G. (2015). Conceptualizing Violence for Health and Medical Geography. Soc. Sci. Med..

[14] Whittle H.J., Palar K., Hufstedler L.L., Seligman H.K., Frongillo E.A., Weiser S.D. (2015). Food Insecurity, Chronic Illness, and Gentrification in the San Francisco Bay Area: An Example of Structural Violence in United States Public Policy. Soc. Sci. Med..

[15] Kearns A., Mason P. (2013). Defining and Measuring Displacement: Is Relocation from Restructured Neighbourhoods Always Unwelcome and Disruptive?. Hous. Stud..

[16] Schnake-Mahl A.S., Jahn J.L., Subramanian S.V., Waters M.C., Arcaya M. (2020). Gentrification, Neighborhood Change, and Population Health: A Systematic Review. J. Urban Health.

[17] Ding L., Hwang J., Divringi E. (2016). Gentrification and Residential Mobility in Philadelphia. Reg. Sci. Urban Econ..

[18] Bhavsar N.A., Kumar M., Richman L. (2020). Defining Gentrification for Epidemiologic Research: A Systematic Review. PLoS ONE.

[19] Maciag M. (2015). Gentrification in America. Governing.com. https://www.governing.com/archive/gentrification-in-cities-governing-report.html.

[20] Richardson J., Mitchell B., Franco J. (2019). Shifting Neighborhoods: Gentrification and Cultural Displacement in American Cities.

[21] Prince S. (2019). Washington D.G.: The District of Gentrification.

[22] Holt S.L., del Río-González A.M., Massie J.S., Bowleg L. (2020). “I Live in This Neighborhood Too, Though”: The Psychosocial Effects of Gentrification on Low-Income Black Men Living in Washington, D.C. J. Racial Ethn. Health Disparities.

[23] Ruiz M.S., O’Rourke A., Allen S.T. (2016). Using Capture-Recapture Methods to Estimate the Population of People Who Inject Drugs in Washington, DC. AIDS Behav..

[24] Aidala A.A., Wilson M.G., Shubert V., Gogolishvili D., Globerman J., Rueda S., Bozack A.K., Caban M., Rourke S.B. (2016). Housing Status, Medical Care, and Health Outcomes Among People Living With HIV/AIDS: A Systematic Review. Am. J. Public Health.

[25] Omerov P., Craftman Å.G., Mattsson E., Klarare A. (2020). Homeless Persons’ Experiences of Health- and Social Care: A Systematic Integrative Review. Health Soc. Care Community.

[26] Allen S., Ruiz M., O’Rourke A. (2015). How Far Will They Go?: Assessing the Travel Distance of Current and Former Drug Users to Access Harm Reduction Services. Harm. Reduct. J..

[27] Government of the District of Columbia, Executive Office of the Mayor Mayor Bowser Announces Homelessness Is Down 47% Since the Implementation of Homeward DC, Hits 17-Year Low. https://mayor.dc.gov/release/mayor-bowser-announces-homelessness-down-47-implementation-homeward-dc-hits-17-year-low.

[28] Cirruzzo C. D.C.’s Struggle to End Homelessness Is Getting More Complicated. https://www.axios.com/local/washington-dc/2022/05/19/dc-aging-homeless-issues.

